# Sudden death associated with silent myocardial infarction in a 35-year-old man: a case report

**DOI:** 10.1186/s13256-016-0823-9

**Published:** 2016-02-29

**Authors:** Mohammad Reza F. Aghdam, Aleksandar Vodovnik, Bjørn Ståle Sund

**Affiliations:** Department of Pathology, Førde Central Hospital, Førde, Norway

**Keywords:** Silent myocardial infarction, Sudden death, Autopsy, Young age, Coronary heart disease

## Abstract

**Background:**

Silent myocardial infarction relates to the absence of symptoms usually associated with myocardial ischemia. It has been estimated that silent myocardial infarction can occur in 2–4 % of young adult asymptomatic men. A majority of patients without an initially apparent cause of sudden death have been found at autopsy to have had significant coronary heart disease, including old, undetected myocardial infarction. Cases of sudden death in young men with unrecognized silent myocardial ischemia seem to be underreported, however.

**Case presentation:**

A 35-year-old Norwegian man without a previous medical history died suddenly without preceding symptoms of coronary ischemia. Apart from elevated lactate, his laboratory test results were within normal limits. An autopsy revealed advanced coronary artery thrombosis of the left anterior descending branch with an extensive, partly organized myocardial infarction. The results of toxicological examinations of peripheral blood were negative for usual narcotics and alcohol.

**Conclusions:**

Sudden, unexpected death due to myocardial infarction can occur even at a young age in patients without known coronary heart disease.

## Background

Cardiovascular disease is the most frequent cause of death worldwide [1]. The first manifestation of coronary artery disease in 60–70 % of patients is either sudden death or myocardial infarction (MI) [2]. Silent myocardial infarction (SMI) relates to absence of symptoms usually associated with myocardial ischemia. Its risk factors include heavy smoking, family history of heart disease, age, high blood cholesterol and systemic blood pressure, diabetes, and overweight [3, 4]. Silent myocardial ischemia may occur in patients of all ages with a coronary disease. It has been estimated that SMI can occur in 2–4 % of young adult asymptomatic men [5]. Previous studies have shown the relationship between increased degrees of pulmonary congestion and painless (not silent) MI in older patients [6]. A majority of patients between the ages of 25 and 60 years without an initially apparent cause of death were reported to have had a significant coronary heart disease at autopsy, including old, undetected MI [7]. Sudden death in young men with silent myocardial ischemia without established risk factors is probably underreported, however.

## Case presentation

**Fig. 1:**
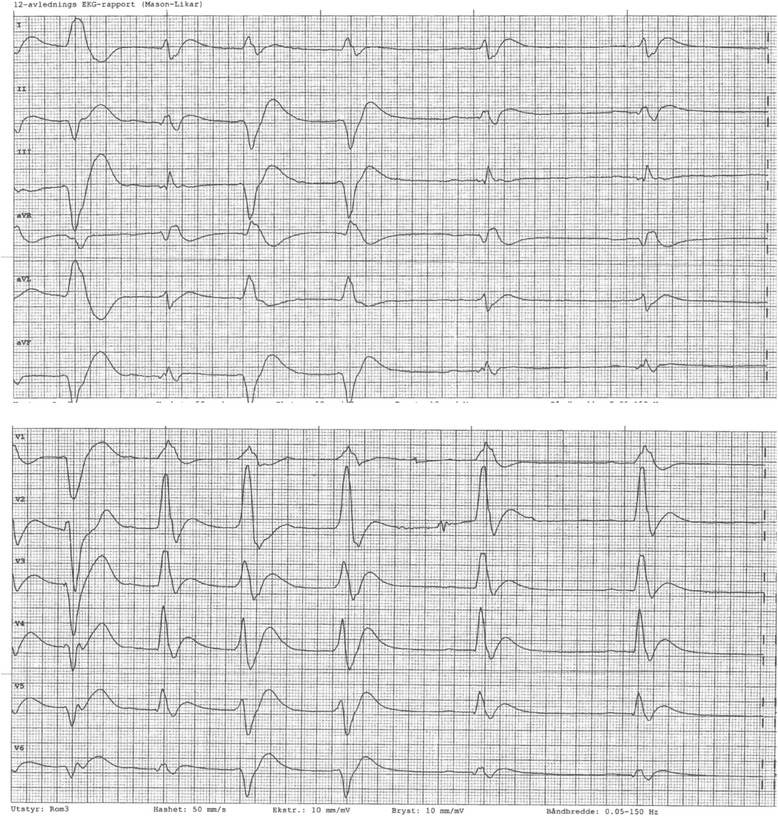
Twelve-lead electrocardiogram shows irregular rhythm with right and left bundle branch block

Our patient was a 35-year-old Norwegian man who suddenly collapsed in the street. Emergency services attended and started cardiopulmonary resuscitation on site, eventually achieving return of spontaneous circulation. The patient experienced another cardiac arrest in the emergency room at the hospital and was given thrombolytic treatment, but he died shortly thereafter. A 12-lead electrocardiogram showed irregular rhythm with both left and right bundle branch block (Fig. 1). Apart from elevated lactate, his laboratory test results were normal (Table 1). According to the patient’s relatives, he had been healthy without known risk factors or a family history of coronary disease. He had occasionally smoked cannabis and hashish and drank alcohol. The use of other narcotic substances was unknown to his relatives or his general practitioner. The patient had been of medium build with a body mass index of 25 kg/m^2^. He had experienced slight chest discomfort 1 month before his death; that episode lasted a couple of hours. The autopsy revealed a 20-mm-long thrombus lodged in the left anterior descending (LAD) branch of the coronary artery associated with moderate atherosclerosis in the same segment. The rest of his vascular system showed only focal mild atherosclerosis. His heart weighed 350 g and was not enlarged. The posterior wall of the left ventricle and part of the septum showed a sharply demarcated infarcted area with a largest diameter of 8 cm. It was yellow-red, soft, and had reddish edges. The rest of the heart was unremarkable. The left and right ventricle walls were 10 and 5 mm thick, respectively.

**Table 1:** Laboratory results related to main and secondary diagnoses

Test	Result	Reference range
Na^+^	136	137–145
K^+^	3.8	3.5–5.0
Cl^−^	99	97–107
Ca^2+^	1.24	2.15–2.55
Hematocrit	47	40–50
Lactate	8.5	0.5–1.6

**Fig. 2:**
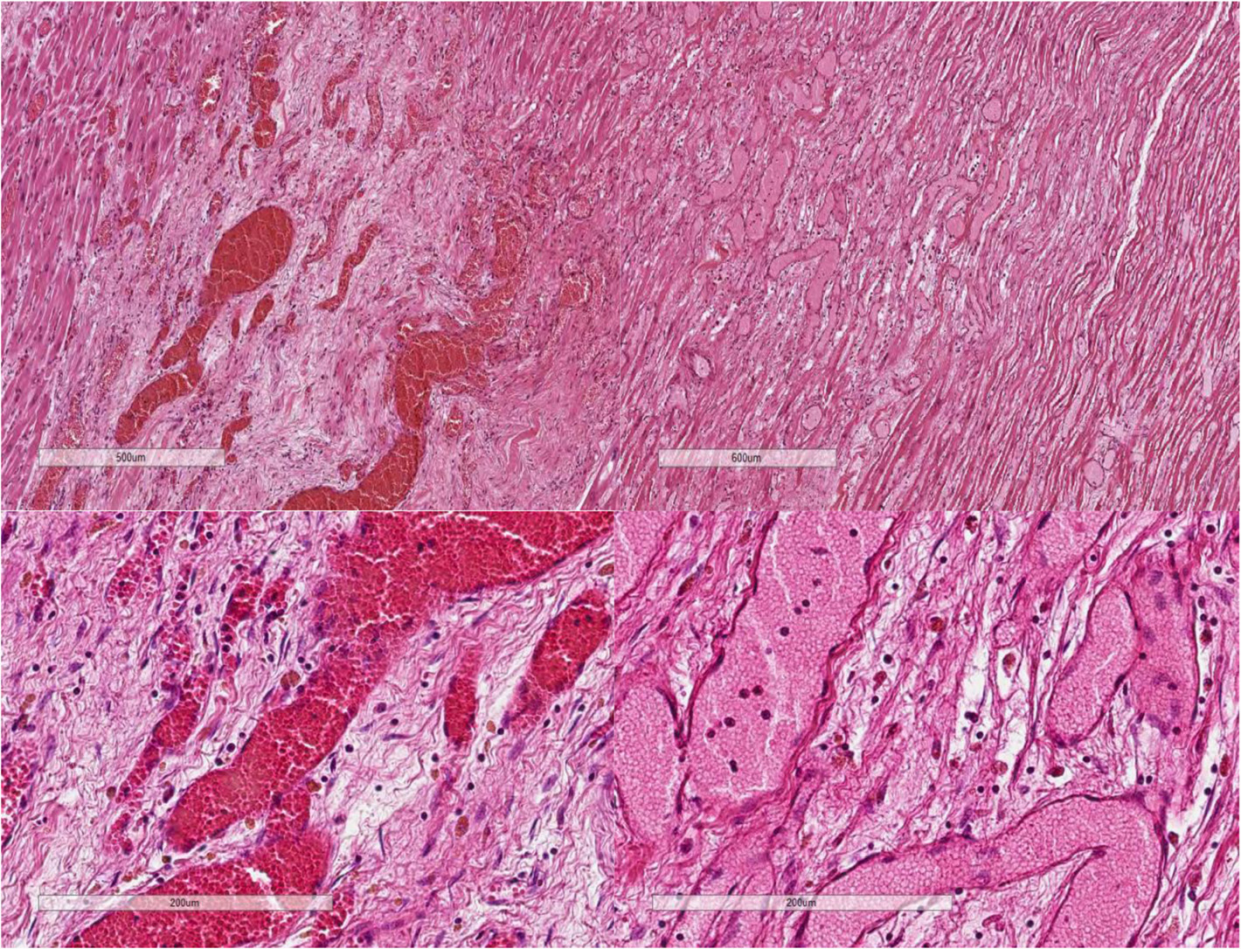
Hematoxylin and eosin–stained section of myocardium illustrating areas of myocardial infarction, early organization (7–10 days old) and acute reinfarction

**Fig. 3:**
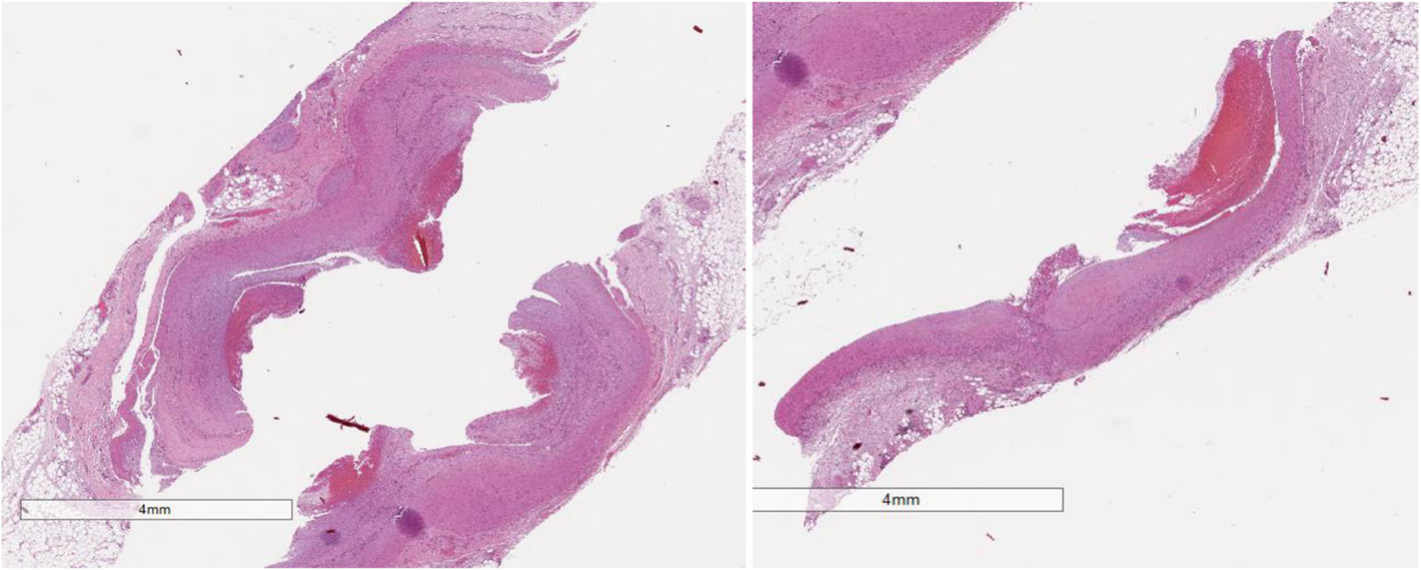
Hematoxylin and eosin–stained section of left anterior descending branch of the coronary artery illustrating areas of atherosclerosis and partly organized thrombosis in the lumen

Other autopsy findings were the main diagnoses of acute MI and thrombosis of the LAD branch of the coronary artery as well as the secondary diagnoses of pulmonary atelectasis; blood stasis in the liver, spleen, and kidney; and mild focal atherosclerosis in the aorta and its major branches. The histological specimens from the heart and LAD branch were fixed in 4 % formaldehyde, embedded in paraffin, and cut at 4 μm. Sections were stained with hematoxylin and eosin. Sections from the heart showed infarction with early organization (7–10 days old) and acute reinfarction at the edges (Fig. 2). At 7–10 days old, an infarction is maximally yellow-tan and soft, with depressed red-tan margins and well- developed phagocytosis of dead cells as well as granulation tissue at the margins [8]. The LAD branch showed atherosclerotic changes in the wall with remnants of a thrombus attached to the intima (Fig. 3).

0

## Discussion

In this case report, we describe sudden death caused by acute MI without other symptoms of coronary ischemia in a young Norwegian man without known risk factors. The findings were compatible with SMI. A previous episode of chest discomfort might have been an early warning sign, even though the patient himself had ignored it as such. Silent myocardial ischemia may occur in patients of all ages with coronary disease, and a majority of patients with SMI who die suddenly are found at autopsy to have had a significant coronary heart disease. Cases of SMI-related sudden death in younger individuals may be underreported in autopsy series worldwide. Knowledge of this disease is crucial with respect to early detection and possible prevention of death. We believe this case report contains a worthwhile clinical lesson for both clinicians and pathologists.

## Conclusions

As presented in our report, SMI with sudden death can occur in young patients and in patients without known risk factors such as a positive family history, regular cigarette smoking, or use of vasoconstrictive drugs. Further autopsy studies on SMI in young individuals are encouraged, and clinical awareness of the condition is mandatory in early intervention to at best avoid a fatal outcome of the disease.

## Consent

Patient's relatives have, in accordance with Norwegian autopsy legislation, given full consent for autopsy including handling of biological material and its use in teaching and research. This is documented by an autopsy requisition form filled out and signed by the patient’s physician at the hospital. A scanned copy of this schema is retained in the patient data base of the hospital; this copy is available for review by Editor-in-chief of this journal.

## Appendix

### List of toxicological analysis (Department of Forensic Toxicology, Norwegian Institute of Public Health)

- Acetone and alcohols (acetone, ethanol, isopropanol, methanol)
- β-Hydroxybutyrate
- Amphetamines (amphetamine, methamphetamine, 3,4-methylenedioxy-methamphetamine, *para*-methoxy-*N*-methylamphetamine, *para*-methoxyamphetamine, meta-chlorophenylpiperazine, 2-(4-bromo-2,5-dimethoxyphenyl)ethanamine, methylenedioxypyrovalerone)
- Cannabis (tetrahydrocannabinol)
- γ-Hydroxybutyrate
- Cocaine (cocaine, benzoylecgonine)
- Opiates/opioids (buprenorphine, ethylmorphine, fentanyl, hydromorphone, codeine, methadone, morphine, oxycodone)
- Benzodiazepines and benzodiazepine derivatives (alprazolam, bromazepam, diazepam, *N*-desmethyldiazepam, phenazepam, flunitrazepam, 7-aminoflunitrazepam, clonazepam, 7-aminoclonazepam, lorazepam, midazolam, nitrazepam, 7-aminonitrazepam, oxazepam, zolpidem, zopiclone)
- Analgesics (dextropropoxyphene, ketamine, ketobemidone, paracetamol, salicylic acid, tramadol)
- Antidepressives (amitriptyline, citalopram, doxepin, fluvoxamine, fluoxetine, clomipramine, mianserin, mirtazapine, moclobemide, nefazodone, nortriptyline, paroxetine, reboxetine, sertraline, trimipramine, venlafaxine)
- Antipsychotics (amisulpride, dixyrazine, flupentixol, haloperidol, chlorpromazine, chlorprothixene, clozapine, levomepromazine, olanzapine, perphenazine, quetiapine, risperidone, zuclopenthixol)
- Antiepileptics (phenobarbital, phenytoin, carbamazepine, lamotrigine, pregabalin)
- Others (alimemazine, carisoprodol, meprobamate, methylphenidate, orphenadrine, promethazine, theophylline)

## Abbreviations

LAD: left anterior descending
MI: myocardial infarction
SMI: silent myocardial infarction
