# Correction: Sudden death associated with silent myocardial infarction in a 35-year-old man: a case report

**DOI:** 10.1186/s13256-018-1613-3

**Published:** 2018-02-22

**Authors:** Mohammad Reza F. Aghdam, Aleksandar Vodovnik, Bjørn Ståle Sund

**Affiliations:** Department of Pathology, Førde Central Hospital, Førde, Norway

## Correction

In the publication of this article [1], there is an error in Table 1 at the Test Ca^2+^ at the Result.

The error in Test Ca^2+^ Result: ‘1.24‘

Should instead read Test Ca^2+^ Result: ‘2.24‘

This has now been included in this erratum.
